# Habitat Associations Shape Phlebotomine Sand Fly Assemblages at the Andes–Amazon Interface in Southeastern Peru

**DOI:** 10.3390/biology15100795

**Published:** 2026-05-16

**Authors:** Sergio Méndez-Cardona, Juliana A. Morales-Monje, Alejandro Lopera-Toro, Adrian Forsyth, Alexandra J. Bauer, Olivia R. Magaletta, Panpim Thongsripong, Olga L. Cabrera-Quintero

**Affiliations:** 1 Florida Medical Entomology Laboratory, Department of Entomology and Nematology, Institute of Food and Agricultural Sciences, University of Florida, Vero Beach, FL 32962, USA; 2 Grupo de Entomología, Instituto Nacional de Salud, Bogota 111321, Colombia; 3 Andes Amazon Fund, Washington, DC 20009, USA

**Keywords:** Phlebotominae, leishmaniasis, habitat associations, post-agricultural landscapes, vector ecology

## Abstract

Phlebotomine sand flies remain poorly studied in southeastern Peru, a region with a high burden of cutaneous leishmaniasis (CL). Using modified ultraviolet (UV) light traps, we surveyed sand fly assemblages at Manu Biological Station during the wet season within secondary forest, *Guadua* bamboo forest, fruit crop plots, and peridomicile habitats. We collected 2641 sand flies representing 32 species. Habitat type was the primary driver of assemblage composition, with minimum nightly temperature as the strongest environmental correlate. Sand fly abundance was highest in secondary forest (*n* = 921) and peridomicile habitats (*n* = 836), where assemblages were dominated by *Nyssomyia shawi*, a generalist species. Although *Guadua* bamboo forests harbored lower abundance (*n* = 386), potential vector species comprised 92% of the assemblage compared to 42–86% in other habitats, suggesting that expanding bamboo forests may favor a higher proportion of potential CL vectors. Peridomicile assemblages consisted largely of generalist species that overlapped with adjacent forested habitats, indicating potential pathways for sylvatic-to-peridomestic spillover. Although the study’s limited scope (i.e., limited to a single season and locality) does not allow broad generalization, our findings suggest the importance of habitat in structuring patterns of potential vector exposure.

## Simple Summary

Leishmaniasis is a disease caused by parasites and spread through the bites of infected sand flies, and it remains a health concern in southeastern Peru. However, we still do not fully understand how environmental conditions affect where these insects live in the region. In this study, we collected sand flies during the wet season from four types of habitats, including secondary forests, fruit-crops, bamboo-dominated areas, and around homes, to better understand where they are most common and how local microclimate influences their distribution. We found that sand flies were most abundant in secondary forests and near homes, where some species were also found indoors. Although bamboo forests had fewer sand flies overall, most of the species found there are potentially capable of spreading the disease, making these expanding areas important for future disease monitoring. We also found that changes in nighttime temperature influenced which species were present. These results show that changes in land use and climate may affect where vector proportions or abundance are highest, helping guide more effective monitoring and prevention efforts to protect local communities.

## 1. Introduction

Leishmaniasis ranks among the ten most significant neglected tropical diseases [1] and remains a major public health concern across 99 endemic countries and territories. Cutaneous leishmaniasis (CL) accounts for a substantial portion of this burden, with approximately 83% of the global cases in 2024 reported in just seven countries: Afghanistan, Algeria, Brazil, Colombia, Iran, Peru, and the Syrian Arab Republic [2]. *Leishmania* parasites are transmitted by female phlebotomine sand flies (Diptera: Psychodidae), which are obligate blood feeders. While 549 sand fly species have been recorded in the New World [3], only 54 are recognized as proven or potential vectors [4,5].

In Peru, CL is endemic and persistent, with 4850 and 3667 cumulative cases reported as of epidemiological week 52 in 2024 and 2025, respectively [6]. Despite its epidemiological importance, ecological and diversity studies of phlebotomine sand flies in Peru remain limited. A total of 175 sand fly species have been recorded nationally, including 70 reported from the Cusco region [1,7]. Research within the Manu Biosphere Reserve, which borders the Madre de Dios department, has been restricted mainly to taxonomic surveys and a few seasonal abundance studies [7,8,9,10]. To date, however, no systematic investigation has evaluated sand fly distribution across habitat types in this region of Peru, leaving a critical gap in our understanding of their ecological associations and potential roles in disease transmission.

Elsewhere in the Neotropics, habitat type and the degree of preservation have been shown to influence sand fly assemblages by altering species composition, richness, and abundance [11]. Disturbance-driven shifts in sand fly community structure often arise from changes in vegetation, microclimate, and host availability, which can favor generalist or potential vector species [12]. Such ecological restructuring can, in turn, affect disease dynamics by increasing human–vector contact or expanding the ecological range of competent *Leishmania* vectors. In southern Peru, climatic and environmental factors, particularly annual rainfall and the presence of humid forests, are considered key determinants of leishmaniasis incidence [13]. Deforestation for logging, agriculture, grazing, road construction, and urban expansion has been associated with changes in sand fly density and elevated transmission risk [14]. In the context of accelerating land-use change, leveraging environmental gradients and niche modeling to predict risk shifts under changing climate scenarios is essential for delineating the ecological limits of sand flies and anticipating future transmission [15].

Manu Biological Station, with its documented history of leishmaniasis and its historical importance as a coca-growing estate under the Inca Empire and a late 19th-century farm known as Villa Carmen, provides an ideal setting to examine how sand fly assemblages are structured across habitats experiencing varying degrees of anthropogenic disturbance. This study offers the first systematic evaluation of habitat-specific sand fly communities in southeastern Peru. We test the hypothesis that sand fly assemblage composition and diversity vary across habitat types. Specifically, we (1) characterize species–habitat associations and indicator species across habitat types; (2) identify species occurring within and around human dwellings (intra- and peri-domicile, respectively); and (3) evaluate the influence of microclimatic variables on assemblage structure.

This study integrates ecological, spatial, and environmental analyses to reveal the dominant role of habitat in structuring sand fly assemblages and highlights the unique profile associated with bamboo-dominated forests. These findings establish a baseline for understanding habitat-driven vector dynamics and provide a framework for improving risk assessments and surveillance strategies for CL in the southwestern Amazon.

## 2. Methods

### 2.1. Study Site

Manu Biological Station, formerly known as Villa Carmen (N 12.8955, W 71.4038), is located in the buffer zone of the Manu National Park in southeastern Peru. Past agricultural activities have transformed the area around the station into a mosaic of habitats with varying degrees of human intervention, including secondary forest fragments, *Guadua* bamboo-dominated patches, fruit crop plots, and human dwellings, which we categorized into intradomicile (indoor) and peridomicile (the area immediately surrounding the home). This heterogeneous landscape covers approximately 3622 ha. The station experiences a humid tropical climate, with an annual precipitation of approximately 4000 mm and distinct dry (August) and wet (November–March) seasons. Yearly mean temperature is 22 °C, reaching up to 32 °C on sunny days and dropping to 10 °C during “friajes” (cold fronts) [16].

### 2.2. Entomological Sampling

**Figure 1:**
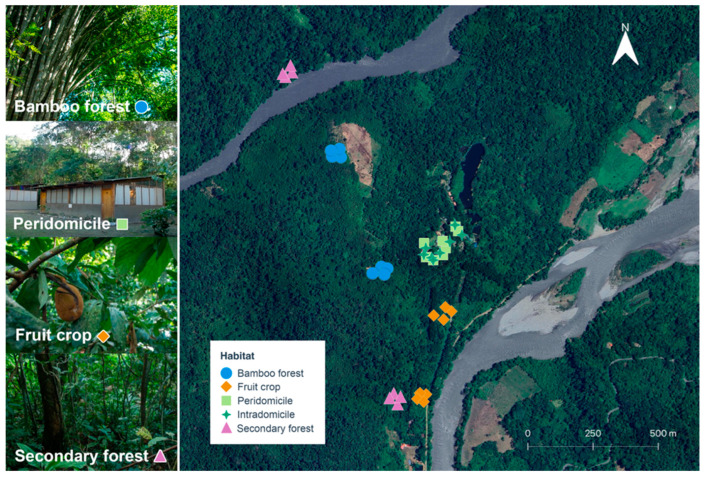
Habitat types and sampling points where sand flies were collected in Manu Biological Station, Cusco, Peru. Shapes represent habitat type. Background imagery: © Google, Maxar Technologies, Airbus, accessed via QGIS v.3.34.12.

To assess differences in sand fly assemblages, we stratified sampling across four habitat types: secondary forest, *Guadua* bamboo forest, fruit crop plots, and peridomicile. We employed a balanced design with two replicate sites per habitat (Figure 1). In the three non-domicile habitats, two spatially distinct sites were selected, and one modified Katchy UV light trap (Katchy, Walpole, MA, USA) [7] was deployed at each site. Hereafter, we refer to this trap as the Manu Micro Trap (MMT). Briefly, the MMT consists of an original UV Katchy suction trap modified with a portable 5 V power system, a rain cover and an external collection bag. This specific configuration was chosen for its portability and high performance in capturing sand flies in Amazonian settings, as detailed in Méndez-Cardona et al. [7]. For the peridomicile habitat, which was restricted to a single settlement, two separate buildings within the cluster were designated as replicate sites to maintain the balanced design. To evaluate sand fly penetration into buildings, paired MMTs were deployed at each building: one placed intradomicile (indoors) and one peridomicile (outdoors).

A total of 10 MMT were operated concurrently (six non-domicile traps and four domicile traps). Traps were active from 18:00 h to 06:00 h the following day. To capture local variation and minimize position bias, traps were relocated each sampling night. Non-domicile traps were moved approximately 10 m within their respective sites, while domicile trap pairs were moved to different buildings within the cluster, while maintaining the indoor/outdoor pairing.

Sampling events lasted for a total of four nights, with each event occurring every 2 weeks over 2 months, from March to April 2023 (wet season). After each event, the traps were placed in plastic bags containing cotton balls soaked in ethyl acetate to immobilize specimens. Collected sand flies were then sorted and preserved in 70% ethanol for transport. In the laboratory, specimens were cleared in 10% potassium hydroxide (KOH) and fixed in saturated phenol for morphological identification following the taxonomic keys of Galati (2018) [17]. Vouchers were deposited at the entomological collection of the National Institute of Health of Colombia in Bogota, Colombia.

### 2.3. Environmental Data Collection

Microclimatic and structural habitat variables were measured at each trap site to assess their influence on sand fly assemblages. Temperature (T) and relative humidity (RH) were recorded at 30 min intervals using dataloggers (Elitech RC-51H USB, San Jose, CA, USA). From these data, we derived six specific microclimatic predictors for each sampling event: minimum temperature (T_min_), mean temperature (T_mean_), and maximum temperature (T_max_) recorded during the 12 h trapping period, as well as T_min24_, T_mean24_, and T_max24_ covering the full 24 h cycle preceding trap retrieval. Similarly, minimum, mean, and maximum relative humidity (RH_min_, RH_mean_, RH_max_) were calculated for the trapping period.

Habitat structure was quantified via foliage cover, crown cover, and crown porosity, estimated from digital cover photography analyzed with the coveR package in R (v.1.1.1) [18]. All photographs were captured at 1.5 m above ground using a Canon PowerShot SX70 HS (Canon Inc., Tokyo, Japan). Additionally, basal area was measured at each trap location using a wedge prism (BAF = 0.46 m^2^/ha) as a proxy for tree density and biomass (Table S1).

### 2.4. Data Analysis

To identify sand fly species associated with specific habitats or combinations of habitats, an indicator species analysis was conducted using the indicspecies package (v1.7.15) in R [19]. Species were identified as indicators based on their Indicator Value (IndVal), which combines measures of specificity (exclusivity to a habitat) and fidelity (frequency of occurrence within that habitat). A species with a significant IndVal (*p* < 0.05) is considered representative of the ecological conditions of its associated habitat(s). Species diversity and richness were estimated with the iNEXT package (v3.0.1), which accounts for differences in sample completeness and allows standardized comparisons across sites and sampling occasions [20]. Singleton species, i.e., those represented by a single individual, were excluded from multivariate analyses to minimize the influence of potential trapping bias and stochastic detection, which can disproportionately affect dissimilarity measures [21,22]. However, singletons were retained for all alpha-diversity and species richness estimates to ensure a representative account of community breadth and to avoid underestimating true species diversity.

Community composition patterns were explored using nonmetric multidimensional scaling (NMDS) based on Bray–Curtis dissimilarity matrices. These analyses were restricted to outdoor assemblages (secondary forest, bamboo, fruit crops, and peridomicile) while intradomicile collections were analyzed separately. NMDS was performed using the metaMDS function in the vegan package (v2.6-8) [23]. We assessed the adequacy of the NMDS solution using ordination stress values, with a threshold of <0.20 considered a usable representation of community patterns [24]. In the resulting two-dimensional ordination plots, each point represents a specific habitat-night sample; proximity between points indicates similarity in sand fly species composition.

To statistically evaluate differences in community structure among habitats and across sampling dates, a permutational multivariate analysis of variance (PERMANOVA) was performed on Bray–Curtis dissimilarity matrices using the adonis2 function in vegan (v2.6-8). Sites were treated as independent replicates, with trap nights averaged to avoid pseudoreplication. The model included habitat type and sampling date as fixed factors, along with their interaction. Homogeneity of multivariate dispersion was tested prior to interpretation using the betadisper function to ensure that significant results reflected differences in community composition rather than within-group variability.

Due to the low number of individuals captured inside homes, intradomicile collections were analyzed separately to characterize relative indoor abundance patterns. For each species, the proportion of females collected indoors was calculated relative to the total number of females captured in the peridomicile area. This proportional metric does not imply habitat preference but rather provides a standardized proxy measure of indoor penetration relative to local outdoor abundance.

All analyses were conducted in R version 4.4.1 [25]. Statistical significance for PERMANOVA and indicator species analysis was assessed at α = 0.05 using 9999 permutations.

## 3. Results

A total of 2641 sand flies, representing 32 species across nine genera, were collected (Table 1). Among the four outdoor habitats, abundance was highest in secondary forest (*n* = 921) and peridomicile environments (*n* = 836), followed by fruit crop plots (*n* = 454) and bamboo forest (*n* = 386). Intradomicile collections yielded only 44 specimens, representing 11 species. Overall, the captures were dominated by females, comprising 74.5% (1969) of the total. This female bias was consistent across habitats: females comprised 82.0% of the peridomicile specimens, 71.9% of the specimens from abandoned crops, 71.5% of the specimens from secondary forests, 62.8% of the specimens from bamboo forests, and 84.1% of the indoor specimens.

### 3.1. Sampling Completeness and Diversity

Sample completeness, estimated as sample coverage, exceeded 0.98 across all habitats, indicating that most individuals in each sample belonged to species that were detected at least once. Rarefaction and extrapolation curves suggested that observed species richness (q = 0) captured 67.1–89.9% of the estimated total richness, with the lowest completeness in peridomicile habitats (25 observed vs. 37.2 estimated species; Table 2). This discrepancy suggests that while abundant species were sampled effectively, rare species remained undersampled.

Species richness (q = 0) was highest in secondary forest (28 species) and peridomicile (25 species). However, Shannon diversity (q = 1), which weights species by their abundance, was highest in fruit crop plots (10.88) and bamboo forests (8.33), and Simpson diversity (q = 2), which emphasizes dominant species, followed the same pattern (Table 2). These contrasting patterns indicate that secondary forest and peridomicile assemblages were dominated by a few highly abundant species, whereas fruit crop and bamboo forest assemblages exhibited greater evenness in species abundances.

### 3.2. Species Composition and Potential Vector Abundance

Species composition differed among habitats. *Nyssomyia shawi* was the dominant species in secondary forest (54%), peridomicile (48%), and bamboo forest (32%), while *Trichophoromyia* spp. dominated in fruit crop plots (29%, Table 2). The number of recognized potential vector species was similar across habitats (10–11 species), but their representation varied markedly (Table 2). Potential vector species comprised the majority of the assemblage in the bamboo forest (92%) and the peridomicile (86%), yet the highest abundances occurred in the secondary forest (737) and the peridomicile (718). Thus, while the bamboo forest exhibited the highest proportional dominance by vectors, the secondary forest and peridomicile harbored the greatest overall vector density.

### 3.3. Species Composition: Intradomicile vs. Peridomicile

Indoor abundances were significantly lower than in the immediate peridomicile, with only 44 individuals collected inside homes. *Nyssomyia shawi* (*n* = 17) and *Psychodopygus llanosmartinsi* (*n* = 14) were the most abundant species found indoors, although they represented only a small fraction of their peridomicile capture (~4 and 11%, respectively). Other species appeared indoors in low numbers: *Psychodopygus yucumensis* (*n* = 4; 10% of peridomicile abundance) and *Trichophoromyia* sp. (*n* = 3; 7%). Single specimens (*n* = 1) were recorded for *Lutzomyia sherlocki*, *Psathyromyia aragaoi*, *Psathyromyia barrettoi*, and *Trichophoromyia macrisae*, while two specimens of *Psychodopygus hirsuta* were identified. Several species common in the peridomicile, including *Evandromyia saulensis*, *Evandromyia walkeri*, and *Psychodopygus carrerai*, were never captured indoors.

### 3.4. Indicator Species and Habitat Associations

**Figure 2:**
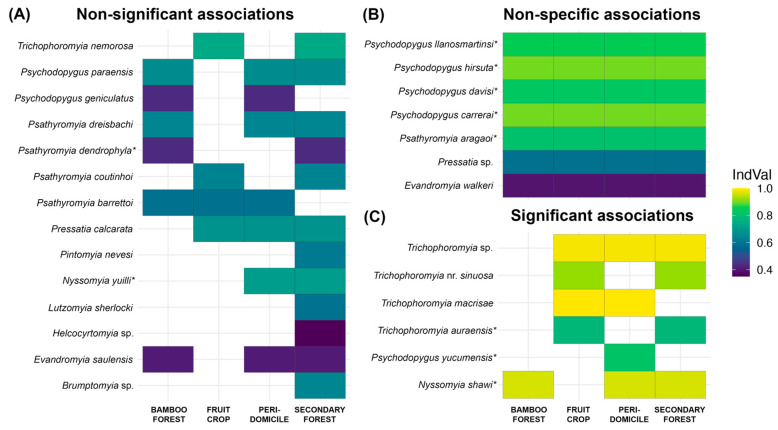
Heatmap depicting habitat associations of sand fly species from multilevel indicator species analysis. Filled cells indicate associations with corresponding habitats, with color intensity representing indicator values (IndVal). Panels show (**A**) non-significant associations (*p* ≥ 0.05), (**B**) non-specific associations (widespread species), and (**C**) significant associations (*p* < 0.05). Asterisks denote potential vectors.

Multilevel pattern analysis identified six indicator species significantly associated with specific habitats or their combinations (Figure 2). Only *Psychodopygus yucumensis* was uniquely associated with a single habitat (peridomicile; IndVal = 0.817, *p* = 0.015). Multiple species showed strong associations with two habitat types: *Trichophoromyia macrisae* with fruit crop plots and peridomiciles (IndVal = 0.995, *p* = 0.005), and two *Trichophoromyia* species (*Tr.* nr. *sinuosa* and *Tr. auraensis)* with fruit crop plots and secondary forest (IndVal = 0.920 and 0.777, respectively; both *p* = 0.005). Two species exhibited broad, three-habitat associations: *Nyssomyia shawi* (IndVal = 0.962, *p* = 0.01) with bamboo forest, peridomicile, and secondary forest, and *Trichophoromyia* sp. (IndVal = 0.987, *p* = 0.005) with fruit crop plots, peridomicile, and secondary forest. No significant habitat associations were detected for the remaining species.

### 3.5. Environmental and Temporal Drivers of Sand Fly Assemblages

**Figure 3:**
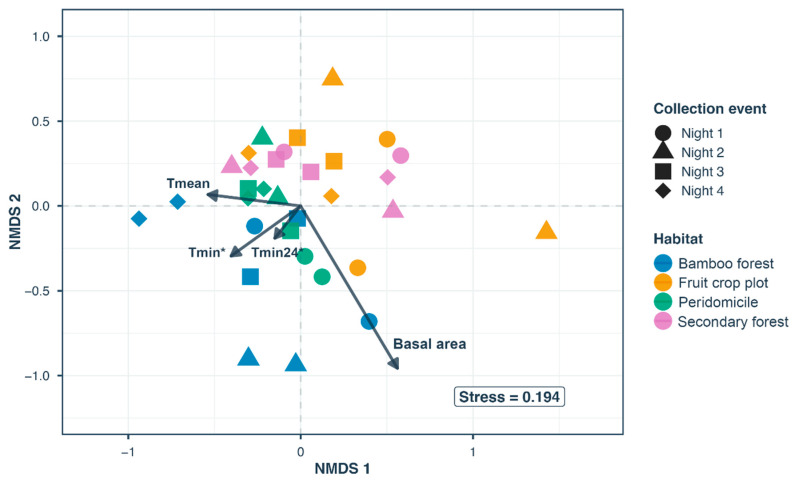
Non-metric multidimensional scaling (NMDS) ordination of sand fly assemblage composition across habitats and sampling dates. Points represent individual collection events, colored by habitat type and shaped by sampling date. Environmental vectors indicate correlates of community composition, with arrow length proportional to correlation strength. Asterisks denote statistical significance (* *p* < 0.05). Stress value indicates the quality of the two-dimensional representation.

PERMANOVA revealed that habitat type was the primary driver of assemblage structure (R^2^ = 0.32, F = 5.29, *p* = 0.0001), with sampling date exerting a weaker but significant effect (R^2^ = 0.14, F = 2.38, *p* = 0.0005). The interaction between habitat and date was not significant (*p* = 0.19), suggesting that habitat associations remained consistent throughout the study period. Tests for homogeneity of multivariate dispersion were conducted to ensure that significant PERMANOVA results reflected differences in assemblage composition rather than differences in within-habitat variability. Dispersion differed among habitats (F = 4.44, *p* = 0.012), driven primarily by higher variability among secondary forest samples. NMDS ordination (stress = 0.194) indicated that sand fly assemblage composition differed among habitat types (Figure 3). Although PERMANOVA detected temporal variation in assemblage composition, sampling dates did not form distinct clusters in ordination space.

Environmental vector fitting identified minimum temperature as the strongest correlate of assemblage composition. Both minimum nightly temperature (T_min_) (R^2^ = 0.26, *p* = 0.012) and minimum temperature in the preceding 24 h (T_min24_) (R^2^ = 0.2, *p* = 0.041) were significantly associated with variation in assemblage composition across habitats (Figure 3). Movement along the T_min_ vector in ordination space corresponded to shifts from peridomicile assemblages toward bamboo forest assemblages, indicating that differences between these habitats were associated with variation in minimum nightly temperature. Mean temperature (T_mean_) (R^2^ = 0.18, *p* = 0.058) and basal area (R^2^ = 0.16, *p* = 0.077) showed marginal associations, whereas vegetation structure variables (foliage cover, crown cover, crown porosity) and humidity were not correlated with community composition (all *p* > 0.1).

## 4. Discussion

Sand fly assemblages in the southwestern Amazon are shaped predominantly by habitat type, with additional influences from temporal and microclimatic variation. The collection of 32 species, along with the new record of *Evandromyia andersoni* for the Cusco Department [26], underscores the continued value of vector surveys in this understudied region. Habitat accounted for 32% of the variation in assemblage composition, while temporal factors explained 14%, indicating that spatial heterogeneity is the primary driver of assemblage structure. Significant differences in multivariate dispersion among habitats further suggest that within-habitat heterogeneity contributes to assemblage differentiation beyond simple shifts in average.

### 4.1. Peridomicile Assemblages and Generalist Vector Species

In ordination space, the peridomicile occupied a central position overlapping with secondary forest, bamboo, and fruit crop habitats (Figure 3), reflecting assemblages largely composed of habitat generalists common in adjacent environments. This pattern could suggest ecological spillover, wherein species from surrounding habitats regularly move into human-modified areas. A notable exception is *Psychodopygus yucumensis*, which showed exclusive association with peridomicile habitats, likely reflecting its anthropophilic behavior [27]. Notably, we observed a low number of captures within the intradomicile. While this limits the strength of conclusions regarding indoor-specific risk, it suggests that human-vector contact in this locality may be primarily peridomestic or occurring at the forest-dwelling interface rather than inside residential structures.

The most abundant species across habitats, *Nyssomyia shawi*, demonstrates the ecological plasticity characteristic of important neotropical vectors such as *Ny. whitmani* in Brazil [28,29]. Indicator species analysis confirmed its significant association with bamboo forest, secondary forest, and peridomicile habitats, and it was the most frequently captured species indoors. This distribution, combined with prior detection of *Leishmania* infections in *Ny. shawi* [30,31], positions this species as a putative bridge vector with the potential to connect peridomestic and sylvatic transmission cycles.

Beyond *Ny. shawi*, other potential vectors may contribute to habitat-specific transmission pathways. *Psychodopygus llanosmartinsi*, the second most common species indoors, displayed a generalist distribution and has been implicated as a vector of *Leishmania* (*Viannia*) *braziliensis* in southeastern Peru [32]. Its frequent occurrence in peridomicile areas indicates substantial peridomestic transmission potential, consistent with observations from lowland sub-Andean Bolivia [27]. Although female *Trichophoromyia* could not be identified to species, the presence of *Th. auraensis*, a putative vector of *L.* (*V.*) *braziliensis* [33,34], suggests additional transmission risk in secondary forests and fruit crop plots where these species were most abundant. Together, habitat-specific assemblage profiles, shaped by the ecological plasticity of potential vector species, have the potential to create distinct transmission pathways and mirror broader patterns of vector dominance in disturbed Neotropical environments [29,35,36,37].

### 4.2. Bamboo Forests: An Emerging Transmission Landscape

*Guadua* sp. dominated forests are rapidly expanding across the region following deforestation, fire, and land abandonment [38,39,40]. We found that bamboo habitats support lower sand fly richness and abundance than secondary forests but maintain higher species diversity and evenness. Notably, potential vectors constituted 92% of the bamboo assemblage, even as the dominant *Ny. shawi* decreased in relative frequency (32%). This reduced dominance suggests that bamboo forests may provide suboptimal conditions for this otherwise ubiquitous species, potentially due to restricted breeding substrates, altered soil–litter properties, or reduced mammal-host availability [41,42]. Simultaneously, the presence of multiple vector species at moderate densities indicates that resilient generalists may successfully colonize these expanding habitats. Although we did not assess *Leishmania* infection in sand flies, the proportional dominance of putative vectors in expanding bamboo forests warrants targeted parasitological surveillance, as increasing human overlap may represent a growing and underappreciated potential for vector-human contact.

### 4.3. Environmental Drivers of Sand Fly Assemblages

Temperature affects both vector survival and *Leishmania* development [43,44], suggesting that fine-scale thermal variation modulates habitat-specific transmission. While structural variables like canopy cover and basal area were weak predictors, minimum nightly temperature (T_min_) and the 24 h minimum (T_min24_) were significantly associated with assemblage composition. Critically, in our ordination analysis, the T_min_ vector was oriented toward bamboo forest and peridomicile assemblages (Figure 3), indicating that variation in minimum temperature corresponded to differences between these habitats and the cooler secondary forest sites.

This pattern is consistent with microclimatic differentiation among habitat types, potentially creating environmental gradients that favor species with differing thermal tolerances [45]. With ongoing warming trends documented across the Amazon basin [46], shifts in minimum temperatures could alter species distributions, potentially enhancing vector persistence in currently marginal habitats such as bamboo forests and expanding the geographic scope of transmission risk.

### 4.4. Study Limitations

This study has several limitations. First, collections were restricted to a two-month period during the wet season due to logistical and resource constraints. Consequently, our findings represent a season-specific snapshot of the sand fly assemblages and likely underrepresent the inter-annual and seasonal variation characteristic of Amazonian populations [9]. Second, the spatial replication was limited to two sites per habitat, which constrains the broader generalizability. At each site, we utilized a concurrent rotation design to capture within-site heterogeneity, relocating traps nightly to generate four unique trapping locations per site. In total, 32 trapping locations provide a snapshot of sandfly communities within the study area, although additional site replication would enhance broader inference. Third, exclusive reliance on modified miniature light traps (MMTs) with UV light-emitting diodes (LEDs) may have introduced detection bias. While UV wavelengths (~365 nm) are highly attractive for many phlebotomines, species-specific differences in spectral sensitivity mean that some taxa may be more attracted to broader incandescent spectra or different wavelengths [7,47,48]. Additionally, our sampling may have missed species that are more abundant in different forest strata (i.e., canopy) [49]. Multi-season sampling and complementary trapping methods would provide a more complete picture of how successional processes and microclimatic gradients shape sand fly assemblages and influence transmission risk in post-disturbance landscapes. Finally, while this study identifies high densities of recognized vector species, we did not assess *Leishmania* infection rates. Consequently, our findings characterize the ecological potential for transmission rather than confirming active parasite circulation in these specific habitats.

### 4.5. Implications for Surveillance and Future Research

Despite these limitations, our findings demonstrate that sand fly assemblage structure is strongly habitat-dependent, creating spatially heterogeneous patterns of potential vector exposure across the landscape. The low dissimilarity of peridomicile assemblages to those found in surrounding habitats, combined with the indoor occurrence of key vector species, highlights the peridomicile–forest interface as a critical zone for vector–human contact. Meanwhile, the high proportional representation of vectors in expanding bamboo forests suggests that landscape transformation may be creating novel transmission pathways that warrant closer epidemiological attention.

Our study provides an empirical foundation for developing habitat-targeted surveillance strategies. We recommend prioritizing two landscape features for monitoring efforts: (1) expanding *Guadua* bamboo forests, where high vector proportions coincide with ongoing habitat expansion and (2) peridomicile areas, where high vector density directly overlaps with human activity. Parasitological screening of dominant vector species, particularly *Ny. shawi* and *Ps. llanosmartinsi*, is essential for confirming their roles in active transmission. Integrated surveillance linking vector distributions, mammalian reservoir hosts, and human case data across habitat gradients will be critical for understanding CL transmission risks in this rapidly changing Amazonian landscape and will prove invaluable for informing future land management and vector control decisions.

## 5. Conclusions

This study, conducted over a two-month period during the 2023 wet season (March–April), demonstrates that habitat type is the primary determinant of sand fly assemblage structure in the southeastern Peruvian Amazon. The significant overlap between peridomestic and secondary forest communities, dominated by the ecologically plastic *Ny. shawi*, highlights a potential pathway for the spillover of *Leishmania* parasites from sylvatic environments into human settlements. Furthermore, we observed a high proportional dominance of potential vector species within expanding *Guadua* bamboo forests. While parasitological confirmation is still required, this ecological trend suggests a potential for pathogen transmission in post-agricultural landscapes. Finally, the identification of minimum nightly temperature as a critical environmental correlate suggests that ongoing climatic shifts may alter vector distributions and persistence. While these findings provide a robust characterization of sand fly assemblages, they represent a specific temporal window (wet season) that warrants further multi-year investigation spanning multiple seasons.

Effective public health interventions in this region should move toward habitat-targeted surveillance strategies. Monitoring efforts should prioritize the peridomicile–forest interface and expanding bamboo patches, where shifts in vector proportional dominance are most likely to occur. To build upon our findings, future research within the region should integrate parasitological screening with multi-season longitudinal sampling to confirm the role of habitat influence and dominant species such as *Ny. shawi* and *Ps. llanosmartinsi* in active transmission cycles. Linking ecological data with human case reports will further improve the ability to anticipate and respond to changing patterns of disease risk across the dynamic Amazonian landscape.

## Figures and Tables

**Table 1:** Sand fly abundance by species and habitat type, with percentage of females per species. Data represent pooled collections across all sampling nights at Manu Biological Station, Peru, March–May 2023.

	Habitat Type						
Species	Intradomicile	Peridomicile	Fruit Crop Plots	Bamboo Forest	Secondary Forest	Grand Total	Proportion Females (%)
*Brumptomyia* sp.		1	1	1	6	9	44.4
*Evandromyia andersoni*				1		1	100
*Evandromyia* n.r. *infraspinosa*		1				1	100
*Evandromyia saulensis*		1		1	2	4	100
*Evandromyia walkeri*		1	1	1	2	5	60
*Helcocyrtomyia* sp.					2	2	50
*Lutzomyia sherlocki*	1	2	1	2	12	18	94.4
Nyssomyia shawi *	17	396	35	123	493	1064	86.4
Nyssomyia yuilli *		9	3	2	28	42	83.3
*Pintomyia nevesi*					3	3	100
*Pintomyia serrana*					1	1	0
*Pressatia calcarata*		5	7		3	15	0
*Pressatia* sp.		4	2	2	4	12	100
*Pressatia triacantha*		1				1	0
Psathyromyia aragaoi *	1	21	19	10	19	70	65.7
*Psathyromyia barrettoi*	1	5	4	4	1	15	13.3
*Psathyromyia coutinhoi*		1	9		4	14	0
Psathyromyia dendrophyla *				2	1	3	100
*Psathyromyia dreisbachi*		17		4	6	27	66.7
*Psathyromyia lutziana*		1				1	100
*Psathyromyia runoides*					1	1	100
Psychodopygus carrerai *		41	30	48	40	159	66.7
Psychodopygus davisi *		16	13	46	39	114	65.8
*Psychodopygus geniculatus*		2		2		4	75
Psychodopygus hirsute *	2	47	37	44	29	159	70.4
Psychodopygus llanosmartinsi *	14	135	29	63	51	292	84.9
Psychodopygus paraensis *		7	5	10	18	40	85
Psychodopygus yucumensis *	4	45	4	6	4	63	85.7
Trichophoromyia auraensis *		1	14		15	30	0
*Trichophoromyia nemorosa*		3	18	6	17	44	0
*Trichophoromyia* sp.	3	45	132	7	78	265	100
*Trichophoromyia macrisae*	1	27	69		1	98	0
*Trichophoromyia* n.r. *sinuosa*		1	21	1	40	63	0
**Grand Total**	**44**	**836**	**454**	**386**	**921**	**2641**	**74.5**

Note: potential vector species are denoted with an asterisk (*).

**Table 2:** Sampling completeness, diversity metrics, and potential vector abundance for sand fly assemblages across habitat types.

Metric	Secondary Forest	Fruit Crop Forest	Bamboo Forest	Peridomicile
**Sampling completeness**				
Sample coverage	0.995	0.993	0.987	0.992
Species coverage	0.871	0.824	0.898	0.671
**Diversity estimates**				
Observed richness (q = 0)	28	21	22	25
Estimated richness	32.2	25.5	24.5	37.2
Shannon diversity (q = 1)	6.9	10.88	8.33	6.81
Simpson diversity (q = 2)	3.26	7.31	5.77	3.77
**Dominant species**	*Ny. shawi* (54%)	*Trichophoromyia* spp. (29%)	*Ny. shawi* (32%)	*Ny. shawi* (48%)
**Potential vectors**				
Proportional abundance	81%	42%	92%	86%
Absolute abundance (*n*)	737	189	354	718

Note: *q* represents the order of Hill numbers: *q* = 0 (Species Richness), *q* = 1 (Shannon Diversity), and *q* = 2 (Simpson Diversity). *n* = absolute abundance.

## Data Availability

Data is contained within the article.

## Supplementary Materials

The following supporting information can be downloaded at: https://www.mdpi.com/article/10.3390/biology15100795/s1, Table S1: Microclimatic and structural habitat variables for sampling sites across habitat types.

## References

[1] World Health Organization (2017). Fact Sheets: Neglected Infectious Diseases—Leishmaniasis.

[2] World Health Organization Global Health Observatory: Leishmaniasis. https://www.who.int/data/gho/data/themes/topics/gho-ntd-leishmaniasis.

[3] Galati E.A.B., de Andrade A.J., Perveen F., Loyer M., Vongphayloth K., Randrianambinintsoa F.J., Prudhomme J., Rahola N., Akhoundi M., Shimabukuro P.H.F. (2025). Phlebotomine sand flies (Diptera, Psychodidae) of the world. Parasit. Vectors.

[4] Killick-Kendrick R. (1999). The biology and control of phlebotomine sand flies. Clin. Dermatol..

[5] World Health Organization (2010). Control of the leishmaniases: Report of a meeting of the WHO Expert Committee on the Control of Leishmaniases, Geneva.

[6] Ministerio de Salud (MINSA) (2025). Boletín Epidemiológico Volumen 34 Semana Epidemiológica 52-2025.

[7] Méndez-Cardona S., Lopera-Toro A., Morales-Monje J.A., Forsyth A., Cabrera-Quintero O.L. (2025). Field evaluation of a commercial light trap for sand fly (Diptera: Psychodidae: Phlebotominae) surveillance in the Peruvian Amazon and new species records for Cusco Department. J. Vector Ecol..

[8] Pérez E., Ogusuku E., Monje J., Young D.G. (1990). *Lutzomyia* (Diptera: Psychodidae) de Pillcopata (Cusco): Nuevos registros para el Perú y descripción de *Lutzomyia deorsa* n. sp.. Rev. Peru. Entomol..

[9] Pérez E., Ogusuku E. (1994). Estacionalidad de *Lutzomyia* spp. (Diptera: Psychodidae) en Coloradito (Pilcopata, Cusco). Rev. Peru. Entomol..

[10] Cáceres A., Quate L., Galati E.A., Baht H. (2001). Flebotomínos (Diptera: Psychodidae) de San Pedro, distrito Kosñipata, Paucartambo—Cusco, y nuevos reportes para el Perú. Rev. Peru. Med. Exp. Salud Publica.

[11] Rebêlo J.M.M., Moraes J.L.P., Cruz G.B.V., Andrade-Silva J., Bandeira M.D.C.A., Oliveira Pereira Y.N., Santos C.L.C.D. (2019). Influence of deforestation on the community structure of sand flies (Diptera: Psychodidae) in Eastern Amazonia. J. Med. Entomol..

[12] de Oca-Aguilar A.M., Rebollar-Téllez E.A., Sosa-Bibiano E.I., López-Avila K.B., Torres-Castro J.R., Loría-Cervera E.N. (2022). Effect of land use change on the phlebotomine sand fly assemblages in an emergent focus of cutaneous leishmaniasis in Yucatan, Mexico. Acta Trop..

[13] Yupari-Azabache I.L., Díaz-Ortega J.L., Bardales-Aguirre L.B., Barros-Sevillano S., Paredes-Díaz S.E. (2023). Cluster analysis of factors associated with leishmaniasis in Peru. Trop. Med. Infect. Dis..

[14] Massey A.L., Ferreira da Silva D.J., Vieira C.J.d.S.P., Allen J.M., Canale G.R., São Bernardo C.S., Bronzoni R.V.d.M., Peres C.A., Levi T. (2025). Using iDNA to determine impacts of Amazonian deforestation on *Leishmania* hosts, vectors and their interactions. PLoS Negl. Trop. Dis..

[15] González C., Wang O., Strutz S.E., González-Salazar C., Sánchez-Cordero V., Sarkar S. (2010). Climate change and risk of leishmaniasis in North America: Predictions from ecological niche models of vector and reservoir species. PLoS Negl. Trop. Dis..

[16] Manu (Villa Carmen) Biological Station | Amazon Conservation Association. https://www.amazonconservation.org/what-we-do/put-science-and-tech-to-work/research-stations/villa-carmen-biological-station/.

[17] Galati E.A.B., Rangel E.F., Shaw J.J. (2018). Phlebotominae (Diptera, Psychodidae): Classification, morphology and terminology of adults and identification of American taxa. Brazilian Sand Flies: Biology, Taxonomy, Medical Importance and Control.

[18] Chianucci F., Ferrara C., Puletti N. (2022). coveR: An R package for processing digital cover photography images to retrieve forest canopy attributes. Trees.

[19] Cáceres M.D., Legendre P. (2009). Associations between species and groups of sites: Indices and statistical inference. Ecology.

[20] Hsieh T.C., Ma K., Chao A. (2016). iNEXT: An R package for rarefaction and extrapolation of species diversity (Hill numbers). Methods Ecol. Evol..

[21] Legendre P., Legendre L. (2012). Numerical Ecology.

[22] Poos M.S., Jackson D.A. (2012). Addressing the removal of rare species in multivariate bioassessments: The impact of methodological choices. Ecol. Indic..

[23] Oksanen J., Simpson G., Blanchet F., Kindt R., Legendre P., Minchin P., O’Hara R., Solymos P., Stevens M., Szoecs E. (2024). *Vegan: Community Ecology Package*, R Package Version 2.6-8. https://CRAN.R-project.org/package=vegan.

[24] Clarke K.R. (1993). Non-parametric multivariate analyses of changes in community structure. Aust. J. Ecol..

[25] R Core Team (2024). R: A Language and Environment for Statistical Computing.

[26] Méndez-Cardona S., Mendieta S., Cabrera-Quintero O.L. (2025). Phlebotomine sand flies (Diptera: Psychodidae) from peridomestic environments at Los Amigos Biological Station, Madre de Dios, Peru. Acta Amazon..

[27] Le Pont F., Desjeux P. (1986). Leishmaniasis in Bolivia: II. The involvement of *Psychodopygus yucumensis* and *Psychodopygus llanosmartinsi* in the selvatic transmission cycle of *Leishmania braziliensis braziliensis* in a lowland subandean region. Mem. Inst. Oswaldo Cruz.

[28] Costa S.M., Cechinel M., Bandeira V., Zannuncio J.C., Lainson R., Rangel E.F. (2007). *Lutzomyia* (*Nyssomyia*) *whitmani* s.l. (Antunes & Coutinho, 1939) (Diptera: Psychodidae: Phlebotominae) and the epidemiology of American cutaneous leishmaniasis in Brazil. Mem. Inst. Oswaldo Cruz.

[29] Valdivia H.O., Zorrilla V.O., Espada L.J., Pérez J.G., Razuri H.R., Vera H., Fernández R., Tong C., Ghersi B.M., Vasquez G.M. (2021). Diversity, distribution, and natural *Leishmania* infection of sand flies from communities along the Interoceanic Highway in the southeastern Peruvian Amazon. PLoS Negl. Trop. Dis..

[30] Ryan L., Lainson R., Shaw J.J. (1987). Leishmaniasis in Brazil. XXIV. Natural flagellate infections of sandflies (Diptera: Psychodidae) in Pará State, with particular reference to the role of *Psychodopygus wellcomei* as the vector of *Leishmania braziliensis braziliensis* in the Serra dos Carajás. Trans. R. Soc. Trop. Med. Hyg..

[31] García A.L., Téllez T., Parrado R., Rojas E., Bermúdez H., Dujardin J.C. (2007). Epidemiological monitoring of American tegumentary leishmaniasis: Molecular characterization of a peridomestic transmission cycle in the Amazonian lowlands of Bolivia. Trans. R. Soc. Trop. Med. Hyg..

[32] Zorrilla V., Vásquez G., Espada L., Ramírez P. (2017). Update on tegumentary leishmaniasis and Carrion’s disease vectors in Peru. Rev. Peru. Med. Exp. Salud Publica.

[33] Valdivia H.O., de los Santos M.B., Fernández R., Baldeviano G.C., Zorrilla V.O., Vera H., Lucas C.M., Edgel K.A., Lescano A.G., Mundal K.D. (2012). Natural *Leishmania* infection of *Lutzomyia auraensis* in Madre de Dios, Peru, detected by a fluorescence resonance energy transfer–based real-time polymerase chain reaction. Am. J. Trop. Med. Hyg..

[34] Teles C.B.G., Santos A.P.D.A.D., Freitas R.A., Oliveira A.F.J.D., Ogawa G.M., Rodrigues M.S., Pessoa F.A.C., Medeiros J.F., Camargo L.M.A. (2016). Phlebotomine sandfly (Diptera: Psychodidae) diversity and their *Leishmania* DNA in a hot spot of American Cutaneous Leishmaniasis human cases along the Brazilian border with Peru and Bolivia. Mem. Inst. Oswaldo Cruz.

[35] Nieves E., Oraá L., Rondón Y., Sánchez M., Sánchez Y., Rojas M., Rondón M., Rujano M., González N., Cazorla D. (2014). Effect of environmental disturbance on the population of sandflies and *Leishmania* transmission in an endemic area of Venezuela. J. Trop. Med..

[36] Ramos W.R., Medeiros J.F., Julião G.R., Ríos-Velasquez C.M., Marialva E.F., Desmouliére S.J.M., Luz S.L.B., Pessoa F.A.C. (2014). Anthropic effects on sand fly (Diptera: Psychodidae) abundance and diversity in an Amazonian rural settlement, Brazil. Acta Trop..

[37] Durán-Luz J., Ibáñez-Bernal S., Rebollar-Téllez E.A., Ibarra-Juárez L.A. (2023). Diversity and spatio-temporal variation of phlebotomine sand flies (Phlebotominae: Diptera: Psychodidae) in three different types of land use and seasons in the state of Veracruz, Mexico. Rev. Mex. Biodivers..

[38] Smith M., Nelson B.W. (2011). Fire favours expansion of bamboo-dominated forests in the south-west Amazon. J. Trop. Ecol..

[39] Virtanen P.K., Apurinã F., Ruokolainen K., Manchineri L. (2022). The role of *Guadua* bamboo in land management and Indigenous perspectives on Bamboo ecosystems in Southwestern Amazonia. Hum. Ecol..

[40] da Silva S.S., Fearnside P.M., de Alencastro Graça P.M.L., Numata I., de Melo A.W.F., Ferreira E.L., de Aragão L.E.O.e.C., Santos E.A., Dias M.S., Lima R.C. (2021). Increasing bamboo dominance in southwestern Amazon forests following intensification of drought-mediated fires. For. Ecol. Manag..

[41] Borges L.H., Calouro A., Botelho A.L., Silveira M. (2014). Diversity and habitat preference of medium and large-sized mammals in an urban forest fragment of southwestern Amazon. Iheringia Ser. Zool..

[42] André C.L., Côrtes M.C., Heming N.M., Galetti M., Alves R.S.C., Bovendorp R.S. (2022). Bamboo shapes the fine-scale richness, abundance, and habitat use of small mammals in a forest fragment. Mammal Res..

[43] Guzmán H., Tesh R.B. (2000). Effects of temperature and diet on the growth and longevity of phlebotomine sand flies. Biomedica.

[44] Hlavacova J., Votypka J., Volf P. (2013). The effect of temperature on *Leishmania* (Kinetoplastida: Trypanosomatidae) development in sand flies. J. Med. Entomol..

[45] Vivero-Gomez R., Duque-Granda D., Rader J.A., Stuckert A., Santander-Gualdron R., Cadavid-Restrepo G., Moreno-Herrera C.X., Matute D.R. (2024). Humidity and temperature preference in two Neotropical species of sand flies. Parasit. Vectors.

[46] Flores B.M., Montoya E., Sakschewski B., Nascimento N., Staal A., Betts R.A., Levis C., Lapola D.M., Esquível-Muelbert A., Jakovac C. (2024). Critical transitions in the Amazon forest system. Nature.

[47] McDermott E.G., Mullens B.A. (2018). The dark side of light traps. J. Med. Entomol..

[48] Zorrilla V.O., Lozano M.E., Espada L.J., Kosoy M., McKee C., Valdivia H.O., Arevalo H., Troyes M., Stoops C.A., Fisher M.L. (2021). Comparison of sand fly trapping approaches for vector surveillance of *Leishmania* and *Bartonella* species in ecologically distinct, endemic regions of Peru. PLoS Negl. Trop. Dis..

[49] Resadore F., Júnior A.M.P., de Paulo P.F.M., Gil L.H.S., Rodrigues M.M.S., Araújo M.S., Julião G.R., Medeiros J.F. (2019). Composition and vertical stratification of phlebotomine sand fly fauna and the molecular detection of *Leishmania* in forested areas in Rondônia State municipalities, Western Amazon, Brazil. Vector Borne Zoonotic Dis..

